# Alternative prediction methods of protein and energy evaluation of pig feeds

**DOI:** 10.1186/s40104-017-0171-7

**Published:** 2017-05-03

**Authors:** Ewa Święch

**Affiliations:** The Kielanowski Institute of Animal Physiology and Nutrition, Polish Academy of Sciences, Jablonna, Poland

**Keywords:** Energy value, In vitro, Pig, Protein value, Rat

## Abstract

Precise knowledge of the actual nutritional value of individual feedstuffs and complete diets for pigs is important for efficient livestock production. Methods of assessment of protein and energy values in pig feeds have been briefly described. In vivo determination of protein and energy values of feeds in pigs are time-consuming, expensive and very often require the use of surgically-modified animals. There is a need for more simple, rapid, inexpensive and reproducible methods for routine feed evaluation. Protein and energy values of pig feeds can be estimated using the following alternative methods: 1) prediction equations based on chemical composition; 2) animal models as rats, cockerels and growing pigs for adult animals; 3) rapid methods, such as the mobile nylon bag technique and in vitro methods. Alternative methods developed for predicting the total tract and ileal digestibility of nutrients including amino acids in feedstuffs and diets for pigs have been reviewed. This article focuses on two in vitro methods that can be used for the routine evaluation of amino acid ileal digestibility and energy value of pig feeds and on factors affecting digestibility determined in vivo in pigs and by alternative methods. Validation of alternative methods has been carried out by comparing the results obtained using these methods with those acquired in vivo in pigs. In conclusion, energy and protein values of pig feeds may be estimated with satisfactory precision in rats and by the two- or three-step in vitro methods providing equations for the calculation of standardized ileal digestibility of amino acids and metabolizable energy content. The use of alternative methods of feed evaluation is an important way for reduction of stressful animal experiments.

## Background

The exact knowledge of the actual nutritional value of individual feedstuffs and complete diets is indispensable for efficient animal production. Therefore, the development of methods determining protein and energy values in pig feeds has always been an important aim of nutritional research.

Research on feed energy values for different animal categories has started very early and since the 19^th^century many energy systems and equations relating energy value to crude or digestible nutrient contents have been developed. On the contrary, well-grounded methods of evaluating feeds as protein sources for monogastric animals that take into account their amino acid (AA) content and ileal digestibility have occurred more recently. In this field, studies showing that only those essential AA, which are absorbed in the small intestine can be utilized by pigs for protein synthesis [1], and the implementation of methods measuring ileal digestibility of protein and AA [2, 3] are the most important achievement. Ileal digestibility of AA are determined based on the difference between dietary AA intake and unabsorbed AA at the terminal ileum, whereas available AA are those absorbed from the gastrointestinal tract in a form suitable for metabolism or protein synthesis [3, 4]. There is no direct measure of AA availability. Standardized ileal digestible AA content may be a good predictor of available AA content in unprocessed feeds and for most AA in processed feeds. However, the available AA content in processed feeds may not always be accurately predicted by ileal digestibility estimates, especially for lysine, methionine, cysteine, threonine and tryptophan [4].

Energy value expressed either as digestible, metabolizable or net energy (DE, ME and NE, respectively) is affected primarily by total tract digestibility of nutrients. Determination of nutrient total tract digestibility in the experiments involving pigs is time-consuming and expensive, while determination of ileal digestibility of protein and AA additionally requires the use of surgically-modified animals. Therefore, more simple, rapid, and animal-sparing methods consistent with the 3R principle (reduction, replacement and refinement), are needed.

Several methods simulating digestive processes in pigs have been recently developed and incorporated into alternative energy and protein evaluation systems. Validation of these methods has been carried out by comparing the results obtained using these methods and in pigs. It has been demonstrated that they have certain limitations that should be considered when applied.

The present study outlines the currently used methods of protein and energy value determination in pigs as well as proposes and describes alternative methods and their advantages.

## Principles of protein and energy value determination in pigs

### Ileal digestibility of protein and amino acids

Protein value of feeds for pigs is defined as the content and proportion of essential AA available for protein synthesis and metabolic purposes. There is general agreement that ileal rather than fecal digestibility measurements represent more accurate estimates of AA availability in pig feeds [1, 5]. The method of ileal digestibility determination consists of the measurement of ileal outflow of protein and AA either by the total collection of digesta or based on the undigested marker content in digesta. Protein and AA digestibility determined in pigs may be expressed as apparent (AID), standardized (SID) or true (TID) digestibility values, depending on how endogenous gut protein and AA losses are considered in the measurement of digestibility [3]. Two types of endogenous losses of protein and AA (EPL and EAAL, respectively) have been distinguished: basal losses defined as minimum losses in relation to the dry matter intake and independent of the dietary composition, and the extra losses caused by the content of fiber or other antinutritional factors [6, 7]. In the AID determination, the total amount of protein and AA in the ileal outflow originating both from undigested feed protein and excreted in the form of EPL/EAAL, are included in the calculations. In contrast, the amount of protein (and AA) excreted in the form of basal EPL and EAAL is calculated and subtracted from total protein (and AA) outflow when determining SID. In consequence, the SID values are higher than those of AID. It was agreed that SID values are more correct than AID to use in diets formulation, as they are more likely to be additive in mixtures of feed ingredients [8]. The TID of protein and AA represents the digestibility of feed protein per se, but since the determination of TID in animals is practically impossible, it is limited to the in vitro methods. The SID of protein and AA can be calculated from AID of protein and AA after adjustment for basal EPL and EAAL, respectively, using following equations [3]:

$$ \begin{array}{l}\mathrm{SID}\;\mathrm{P}=\mathrm{AID}\;\mathrm{P}+\frac{\mathrm{basal}\ \mathrm{EPL}}{\mathrm{P}\ \mathrm{content}}\times 100\\ {}\mathrm{SID}\;\mathrm{AA}=\mathrm{AID}\;\mathrm{AA}+\frac{\mathrm{basal}\ \mathrm{EAAL}}{\mathrm{AA}\ \mathrm{content}}\times 100\end{array} $$

where:

- SID P - standardized ileal digestibility of protein expressed in %,
- SID AA - standardized ileal digestibility of amino acids expressed in %,
- AID P - apparent ileal digestibility of protein expressed in %,
- AID AA - apparent ileal digestibility of amino acids expressed in %,
- basal EPL – basal endogenous protein losses expressed in g per kg of dry matter intake,
- basal EAAL – basal endogenous amino acid losses expressed in g per kg of dry matter intake,
- P content – protein content in feed expressed in g per kg dry matter,
- AA content – amino acid content in feed expressed in g per kg dry matter.

The SID values can be also calculated from TID of protein determined in vitro using equations according to Boisen [9]. These equations are given in section describing in vitro method for prediction of ileal digestibility of protein and AA by Boisen and Fernández [10].

Several methods can be used for measuring ileal protein and AA outflow (including the slaughter technique, various cannulation techniques [11] and ileo-rectal anastomosis [12]) and determination of EPL and EAAL (including protein-free feeding, regression method, enzymatically hydrolyzed protein/ultra-filtration method, homoarginine method and isotope dilution technique). Advantages and disadvantages of these methods were described in details many times, e.g. [7, 13, 14].

### Energy value

The energy value of feeds may be expressed as the content of DE, ME, and NE, in accordance with the following steps of energy utilization by the pig: digestive utilization (energy intake – energy excreted in feces), metabolic utilization (energy digested – energy excreted in urine and gases) and net utilization (energy metabolized – heat losses) [15]. The values of ME and NE are preferably used in many feed evaluation systems. The energy excreted in feces can be measured by the total collection of feces in a conventional balance experiment or by collecting grab samples and using the marker technique. Energy losses in urine are determined by the total collection of urine in a conventional balance experiment or via a catheter in urine bladder. Energy losses in the form of gas are either disregarded or calculated as a function of fermented cell wall content [16]. Animal heat production can be determined by calorimetry (direct or indirect) or based on changes in body composition (slaughter technique).

## Alternative methods applied in feed evaluation

There is a need for simple, inexpensive, rapid and reproducible alternative methods for routine assessment of nutrient digestibility instead of direct measurement in pigs. Nutrient digestibility including ileal digestibility of protein and AA and energy value in pig feeds can be estimated using the following alternative methods: i) prediction equations based on chemical composition; ii) animal models, such as rat, cockerels and growing pigs for adult animals; and iii) rapid methods as the mobile nylon bag technique and in vitro methods.

### Prediction equations based on chemical composition

A number of different equations for calculating the energy value of pig feeds from their chemical composition have been developed and used. One of the oldest and well-documented set of equations was developed as the so-called Rostock system based on the nutrients analyzed using the Weende method [17]. The system was later updated and extended to include more nutrients (starch and sugars) [18]. Another set of equations was proposed by Noblet and Perez [19], who determined the content of DE and ME in 114 pig diets differing widely in chemical composition. It has been reported that satisfactory precision of DE and ME assessment based on chemical characteristics can be achieved using ash, crude protein, ether extract and neutral detergent fiber contents as predictors (Table 1), but several equations comprising other set of components have been also proposed [16].

**Table 1:** Prediction equations of energy values of pig feeds based on their chemical composition [19]

Equations^a^	*R* ^2^
DE = 4477 – 10.0 × Ash + 3.8 × EE - 7.1 × CF	0.82
DE = 4443 – 6.9 × Ash + 3.9 ×EE – 4.0 × NDF	0.88
DE = 4151 – 12.2 × Ash + 2.3 × CP + 3.8 × EE – 6.4 × CF	0.89
DE = 4168 – 9.1 × Ash + 1.9 × CP + 3.9 × EE – 3.6 × NDF	0.92
DE = 1407 + 0.657 × GE – 9.0 × Ash + 1.4 × CP – 6.7 × CF	0.86
DE = 1161 + 0.749 × GE – 4.3 × Ash – 4.1 × NDF	0.91
DE = 949 + 0.789 × GE – 3.5 × Ash – 3.8 × NDF – 5.4 × ADL	0.92
DE = 1007 + 0.750 × GE – 4.6 × Ash + 0.8 × CP – 3.6 × NDF – 5.0 × ADL	0.93
ME = 4369 – 10.9 × Ash + 4.1 × EE - 6.5 × CF	0.87
ME = 4334 – 8.1 × Ash + 4.1 × EE -3.7 × NDF	0.91
ME = 4168 – 12.3 × Ash + 1.4 × CP + 4.1 × EE - 6.1 × CF	0.88
ME = 4194 – 9.2 × Ash + 1.0 × CP + 4.1 × EE – 3.5 × NDF	0.92
ME = 1255 + 0.712 × GE – 8.5 × Ash – 6.6 × CF	0.85
ME = 1099 + 0.740 × GE - 5.5 × Ash – 3.7 × NDF	0.91

^a^ *DE* Digestible energy, *ME* Metabolizable energy, *EE* Ether extract, *CF* Crude fiber, *CP* Crude protein, *GE* Gross energy, *NDF* Neutral detergent fiber, *ADF* Acid detergent fiber, *ADL* Acid detergent lignin, energy values and chemical composition expressed in kcal per kg of DM and g per kg of DM, respectively

*R* ^2^ – coefficient of determination

Various systems of feed energy evaluation based on chemical composition are currently used worldwide in intensive pig production. However, other verification methods of the energy values of feeds produced with new technologies and from non-conventional raw materials are often needed.

In contrast to the energy values, only few equations have been proposed for the prediction of protein and AA ileal digestibility in feedstuffs for growing pigs. Moreover, these equations were calculated using a low sample number of only several feedstuffs. Hall et al. [20] used crude protein, neutral detergent fiber and protein associated with neutral detergent fiber as predictors to estimate AID of protein and AA in eleven different feedstuffs (seven cereals or cereal fractions, two oilseed meals and two animal products). It has been shown that content of crude protein, neutral detergent fiber and nitrogen associated with neutral detergent fiber can be used as predictors (*R* ^2^ = 0.95, 0.93, 0.92 and 0.96, respectively) of AID of protein, lysine, threonine and methionine in feedstuffs for pigs [20]. To my knowledge, there have been only one published study relating SID of protein and AA in feedstuffs to their chemical composition. Février et al. [21] have compared SID of main AA and chemical composition of two oilseed meals and reported that the content of ash, fat, nitrogen, protein associated with neutral detergent fiber and of gossypol were useful as predictors to estimate SID of lysine, threonine and tryptophan (*R* ^2^ = 0.993, 0.983 and 0.959, respectively) only in cotton seed meal, but not in palm kernel meals [21].

No need to sacrifice the animal and conduct laboratory experiments is the advantage of using prediction equations for feed value estimations. However, the validity of prediction of energy and protein values for different feeds based on chemical composition depends on the appropriate choice of equations and accuracy of analytical methods, and may be limited by the presence of factors or processes decreasing the assimilation of nutrients.

### Animal model

Small monogastric animals have been widely used as a model for growing pigs in digestibility studies. Advantages of the experiments on laboratory animals are their low body weight and food intake.

#### Rats

It was shown that the rat is a suitable animal model for pigs in studies on the ileal digestibility of protein and AA [22–28] and total tract digestibility of energy or energy value [29–32] in many single feedstuffs and complete diets. For the determination of ileal digestibility in rats, the slaughter method of digesta collection was used and the ileal digestibility values were compared with those obtained in pigs using T-cannulation [22, 28] or the slaughter technique [23, 24, 27].

The detailed experimental procedures aimed at the optimizing ileal digestibility determination in rats have been studied. The animals are housed individually in metabolic cages in room with controlled temperature and 12-h light/dark cycle. Fresh water is available at all time. The following feeding systems have been used: single meal in the morning [23, 26, 28, 33, 34], few equal meals [24, 27] or ad libitum feeding [22, 25]. Optimum time of sampling after the start of feeding and site in the ileum for collecting digesta have been also investigated [26, 33, 34]. It has been reported that the ileal digestibility of protein was relatively constant over the sampling times of 3-6 h following a meal, but was the least variable 4 h after meal [26, 34]. Ileal digestibility of protein was lower when digesta were taken over 20 cm of distal ileum in comparison with shorter sampling lengths [34] and was similar when digesta were taken from shorter site than distal 20 cm [26]. It has been recommended to collect ileal digesta 4 h post-feeding from the distal 10 cm of ileum [34]. Due to small volume of digesta sampled from each animal, it is necessary to pool samples from few animals or use very sensitive methods of analysis.

In some comparative studies, ileal digestibility of protein and AA in rats and pigs were expressed only as AID [22, 24]. Therefore, for the need of present paper, the AID values for protein and main AA reported for pigs and rats [22, 24] have been recalculated to SID ones and the equations predicting pig SID from rat SID values have been computed (Table 2). Values of basal EPL and EAAL for rat were calculated as mean value from data available in literature [25, 27, 28, 33, 35–42], whereas tabulated values according to Rademacher et al. [43] were taken for pigs (Table 3). Generally, EAAL in rats were lower or similar to tabulated values of EAAL in pigs. Among the main AA, the biggest differences between pigs and rats were obtained for lysine and threonine. A close relationship (*R* ^2^ = 0.759, 0.929, 0.761, 0.887 and 0.947 for protein, lysine, cysteine, threonine and isoleucine, respectively) between SID of protein and main AA (with exception of methionine) determined in pigs and rats have been found (Table 2). Based on presented results, it can be concluded that the laboratory rat can be used as a model for determination of SID of AA in pig feeds, especially lysine, isoleucine and threonine. However, rat assay may not be suitable for all types of feedstuffs. It was shown that the ileal digestibility of protein and AA in pea was higher in rats than in pigs [22, 28] probably due to their smaller sensitivity to tannins present in peas [44].

**Table 2:** Prediction equations of standardized ileal digestibility in pigs from values obtained for rats

Equations^a^	n	*R* ^2^
SID_PIG_ P = 21.822 + 0.70826 × SID_RAT_ P	13	0.759
SID_PIG_ Lys = 1.4407 + 0.98454 × SID_RAT_ Lys	15	0.929
SID_PIG_ Met = 9.7265 + 0.88122 × SID_RAT_ Met	14	0.445
SID_PIG_ Cys = 36.247 + 0.50543 × SID_RAT_ Cys	13	0.761
SID_PIG_ Thr = 13.491 + 0.84369 × SID_RAT_ Thr	15	0.887
SID_PIG_ Ile = 1.0205 + 1.0004 × SID_RAT_ Ile	15	0.947

^a^calculated from results of Moughan et al. [22]; Moughan et al. [23]; Smith et al. [24]; Donkoh et al. [26]; Rutherfurd and Moughan [27]; Święch and Buraczewska [28]; Święch [91]

SID_PIG_ and SID_RAT_ – Standardized ileal digestibility determined in pigs and rats expressed in %, respectively; *P* Protein, *Lys* Lysine, *Met* Methionine, *Cys* Cysteine, *Thr* Threonine, *Ile* Isoleucine

*R* ^2^ – coefficient of determination

**Table 3:** Endogenous protein and amino acids losses in rats and pigs^a^

	Rat^b^					Pig^c^
	Mean	SD	n	Min	Max	Mean
Protein	11.102	1.914	8	6.894	13.300	11.82
Lysine	0.293	0.108	18	0.163	0.522	0.40
Methionine	0.098	0.038	15	0.045	0.157	0.11
Cysteine	0.173	0.102	8	0.056	0.380	0.21
Threonine	0.501	0.149	18	0.278	0.782	0.61
Isoleucine	0.331	0.192	18	0.118	0.803	0.38
Arginine	0.234	0.083	18	0.084	0.398	0.39
Histidine	0.170	0.054	18	0.090	0.307	0.19
Leucine	0.398	0.147	18	0.229	0.706	0.49
Phenylalanine	0.214	0.065	18	0.133	0.330	0.34
Valine	0.392	0.178	18	0.189	0.798	0.54

^a^expressed in g per kg dry matter intake

^b^References: [25, 27, 28, 33, 35–42]

^c^Reference: [43]

Rats have been tested also as a model for predicting energy value of pig feeds. In majority of experiments, apparent total tract digestibility (ATTD) of energy and DE value of single feedstuffs and diets were determined [29–31]. Similar DE values of diets differing in gross energy (3.2 and 3.9 cal/g) and protein content (14% and 18% crude protein) were reported by Likuski et al. [30] and of four cereals by Smith et al. [31]. A close relationship between ATTD of energy and energy value of feedstuffs and diets determined in pigs and rats was also found by Furuya et al. [29], Smith et al. [31], Beames et al. [45] and Jørgensen and Lindberg [32]. Equations describing these relationships are given in Table 4. Higher energy values of high-fiber diets for pigs than rats [46] are probably due to a greater ability of pigs to digest crude fiber, whereas higher DE of oats for rats than pigs [31] may be related to higher digestibility of fat in rodents.

**Table 4:** Prediction equations of energy digestibility and energy values for pigs from values determined for rats

Equations^a^	Samples type	n	*R* ^2^	Reference
DE_PIG_ = –0.702 + 1.183 × DE_RAT_	Diets	16	0.940	[29]
ATTD_PIG_ E = –15.48 + 1.1615 × ATTD_RAT_ E	Cereals	5	0.992	[31]
DE_PIG_ = –4.489 + 1.2532 × DE_RAT_	Cereals	5	0.996	[31]
ME_PIG_ = –5.176 + 1.3015 × ME_RAT_	Cereals	5	0.995	[31]
ATTD_PIG_ E = 0.211 + 0.766 × ATTD_RAT_E	Feedstuffs	138	0.81	[32]
ATTD_PIG_ E = 0.104 + 0.867 × ATTD_RAT_ E	Cereals	56	0.93	[32]
ATTD_PIG_ E = 45.261 + 0.00549 × ATTD_RAT_ E	Barley	18	0.971	[45]

^a^DE_PIG_ and DE_RAT_: Digestible energy for pigs and rats expressed in MJ per kg dry matter, respectively; ATTD_PIG_ E and ATTD_RAT_ E: Apparent total tract digestibility of energy determined in pigs and rats expressed in %; *R* ^2^ – coefficient of determination

It can be concluded that in spite of some anatomical and physiological differences between the two species [47] rat may be a useful model for pigs for estimation of ATTD of energy of feedstuffs and diets with moderate fiber and fat content.

#### Cockerels

Results of experiments on the use of birds as a model for predicting the energy value of pig diets are inconsistent. A close relationship (*R* ^2^ = 0.939) was found between the true ME of five samples of low-fiber cereals determined in cockerels and apparent DE determined in growing pig, whereas the ME values of high-fiber cereal (oat) differed greatly between the two species [48]. Lack of correlation (*R* ^2^ = 0.03) between apparent ME of 39 barleys determined in chickens and DE measured in pigs was reported by Zijstra et al. [49] and a rather low correlation (*R* ^2^ = 0.697) between ME values of 70 feedstuffs and 14 mixed feeds determined in both species was found by Sibbald et al. [50]. The correlations were increased when barley diets fed to birds were supplemented with ß-glucanase (*R* ^2^ = 0.56) [49] and when fiber content was included into the equations (*R* ^2^ = 0.699, 0.858, 0.923 and 0.833 vs. 0.697, 0.775, 0.885 and 0.710, respectively) in the study of Sibbald et al. [50]. Equations describing relationship between ME values determined in cockerels and pigs [50] are presented in Table 5. It is concluded that birds are not a good model for predicting energy value of pig diets.

**Table 5:** Prediction equations of metabolizable energy for pigs from values determined for cockerels [50]

No.^a^	Equation^b^	n	*R* ^2^
1	ME_PIG_ = 4.170 + 0.7405 × ME_COCKERELS_	84	0.697
2	ME_PIG_ = 4.826 + 0.7016 × ME_COCKERELS_ – 0.002464 × CF	84	0.699
3	ME_PIG_ = 4.966 + 0.6924 × ME_COCKERELS_	80	0.775
4	ME_PIG_ = 9.392 + 0.4292 × ME_COCKERELS_ – 0.01535 × CF	80	0.858
5	ME_PIG_ = 3.312 + 0.7924 × ME_COCKERELS_	51	0.885
6	ME_PIG_ = 6.686 + 0.5960 × ME_COCKERELS_ – 0.01190 × CF	51	0.923
7	ME_PIG_ = 4.700 + 0.7634 × ME_COCKERELS_	29	0.710
8	ME_PIG_ = 9.891 + 0.4274 × ME_COCEKRELS_ – 0.01612 × CF	29	0.833

^a^Equations: No. 1 and 2 - estimated for 84 samples (70 feedstuffs and 14 mixed diets); No. 3 and 4 - estimated after exclusion four samples of meat and bone meals; No. 5 and 6 - estimated for cereals and their by-products, vegetable proteins and mixed diets; No. 7 and 8 - estimated for animal protein and miscellaneous

^b^ME_PIG_ and ME_COCKERELS_: Metabolizable energy determined for pigs and cockerels, respectively, expressed in MJ per kg dry matter; CF: Content of crude fiber expressed in g per kg dry matter

*R* ^2^ – coefficient of determination

There have been no data on using cockerels as model for prediction of ileal digestibility of protein and AA in pig diets.

#### Growing pigs as a model for adults

The energy values of many feedstuffs and diets have been compared between growing pigs and adult sows [51–53]. The DE values of 67 diets determined in adult sows and growing pigs were closely related, but were higher in adult sows than in growing pigs. The differences were primarily due to the higher rate of degradation of dietary fiber in the large intestine of sows [51]. A close correlation (*R* ^2^ = 0.92) between DE values of cereals and cereal containing diets determined in adult sows and growing pigs was reported also by Cozannet et al. [52] and between values determined in gestating sows and growing pigs by Lowell et al. [53]. The DE values of all diets were, however, higher for gestating sows than growing pigs. Equations for predicting energy values for adult sows from values obtained for growing pigs are presented in Table 6. It can be concluded that two different energy values of feeds should be used for growing pigs and adult sows during formulation of diets [53].

**Table 6:** Prediction equation of energy values for sows from values obtained for growing pigs

No.^a^	Equations^b^	n	*R* ^2^	Reference
1	DE_ADULT SOW_ = 4.37 + 0.742 × DE_GROWING PIG_	67	0.89	[51]
2	DE_ADULT SOW_ = 0.984 × DE_GROWING PIG_ + 0.0045 × NDF	67	0.90	[51]
3	DE_ADULT SOW_ = 1.012 × DE_GROWING PIG_ + 0.0060 × ADF	67	0.85	[51]
4	DE_ADULT SOW_ = 1.014 × DE_GROWING PIG_ + 0.0066 × CF	67	0.82	[51]
5	DE_ADULT SOW_ = 0.991 × DE_GROWING PIG_ + 0.0036 × fiber	67	0.87	[51]
6	DE_ADULT SOW_ = 3.01 + 0.85 × DE_GROWING PIG_	19	0.92	[52]
7	DE_GESTATING SOW_ = 3.237 + 0.810 × DE_GROWING PIG_	11	0.77	[53]
8	ME_GESTATING SOW_ = 5.080 + 0.672 × ME_GROWING PIG_	11	0.55	[53]

^a^Equations: No. 1–5 evaluated for diets differing in chemical composition, but contain no more than 60 g of ether extract per kg of dry matter; wheat products contain 10 samples of wheat distillers grains with solubles and 9 samples of wheat and wheat milling coproducts; No. 6 evaluated for wheat products; No. 7-8 evaluated for three diets based on corn, wheat or sorghum and eight diets based on a combination of corn and high-protein feedstuff (soybean meal, canola meal, conventional distillers’ dried gains with solubles or low-fat distillers’s dried grains with solubles) or high-fiber feedstuff (corn germ meal, corn bran, wheat middlings or soybean hulls)

^b^DE_ADULT SOW,_ DE_GESTATING SOW_ and DE_GROWING PIG_: Digestible energy for adult sows, gestating sows and growing pigs, respectively, expressed in kcal per kg dry matter; ME _GESTATING SOW_ and ME_GROWING PIG_: Metabolizable energy for gestating sows and growing pigs, respectively, expressed in kcal per kg dry matter; CF, NDF and ADF: Crude fiber, neutral detergent fiber and acid detergent fiber, respectively, expressed in g per kg of dry matter; fiber = 1 – (ash + crude protein + ether extract + starch + sugars), expressed in g per kg dry matter

*R* ^2^ – coefficient of determination

Stein et al. [54, 55] compared ileal digestibility (AID and SID) of protein and AA in three cereals and three protein concentrates determined in growing pigs, lactating sows and gestating sows. It has been shown that lactating sows, and to a lesser extent gestating sows, had a higher AID of most of AA than growing pigs [54], whereas gestating sows had higher SID of most AA compared with growing pigs and lactating sows [55]. It can be concluded that growing pigs might serve as a good model for predicting SID of AA for lactating, but not for gestating sows. However, differences in SID may be rather due to differences in feeding system (free access to feed in growing pigs and lactating sows vs restricted feeding in gestating sows) than the physiological status of animals [55]. The advantage of measuring the energy and protein values in growing pigs instead of adult sows is a lower feed intake and easier handling of younger animals.

### Rapid methods

Rapid methods comprise the mobile nylon bag technique combining in vitro and in vivo assays, in vitro methods involving many different techniques, and near-infrared reflectance spectroscopy.

#### The mobile nylon mobile technique (MNBT)

The MNBT was described for the first time by Sauer et al. [56]. Nylon bags with a small amount of feed are pre-digested in vitro with pepsin and inserted into the duodenum via cannula during feeding time. The bags can be recovered either from the feces to determine the total tract digestibility [56] or from ileal digesta via cannula [57] and from ileo-rectal anastomized pigs [58, 59] to assess ileal digestibility. The advantages of MNBT is a low amount of feed used, fewer animals needed and a relatively short time of analysis. The MNBT appeared to be a promising approach for the rapid determination of the total tract digestibility of protein in pigs [56]. Although the ATTD of protein determined with the MNBT was lower than those determined in pigs, a close relationship (*r* = 0.925) was found between the results obtained using both techniques for 15 feedstuffs [60]. After inclusion of nitrogen-free extract or crude fiber content to regression analyses, the correlation coefficient improved (*r* = 0.949). The study of Thacker and Qiao [61] showed that the modified MNBT can be used for the rapid determination of the ATTD of dry matter and energy in all feedstuffs, whereas it overestimated the ATTD of protein.

The potential use of the MNBT for determination of ileal digestibility of protein and AA has been also studied [57–59], but the results are inconsistent. Yin et al. [58] reported that AID of protein and AA determined with MNBT were higher than those determined with conventional methods, however, significant correlation have been found between AID of protein and AA determined with MNBT and conventional method. The highest accuracy of predicting the AID using MNBT have been obtained for arginine, valine and threonine (*R* ^2^ = 0.90, 0.81 and 0.79, respectively) and the lowest for histidine and aspartic acid (*R* ^2^ = 0.50 and 0.59, respectively). It is partially in agreement with findings of study by Steiner et al. [57], who found higher AID of protein and AA determined with MNBT than with conventional method, but there were no relationship (*r* from 0.05 to 0.33) between AID determined both techniques. In another study by Yin et al. [59], AID of protein and AA determined with MNBT were similar to those of a conventional digestibility study with ileo-rectal anastomized pigs. The potential use of the MNBT for determination of AID of protein and AA is limited, and its accuracy may be affected by many factors, such as sample size, fineness of grinding, antinutritional factors, handling of the retrieved bags, etc. [57, 59]. The decrease of sample weight (from 1.00 to 0.75 and 0.50 g) and of particle size (from 2 and 3 to 1 mm) increased AID of protein and AA determined with MNBT to values obtained in conventional digestibility study on ileo-rectal anastomized pigs. The AID of protein and AA of diets containing trypsin inhibitor was higher when determined with MNBT than conventionally due to not accounting for greater endogenous secretion [59]. It can be concluded that MNBT is promising technique for rapid determination of energy values of pig feeds, however, it may not be appropriate for assessing AID of protein and AA in feeds containing trypsin inhibitor or other antinutritional factors that increase EPL and EAAL [57, 59].

#### In vitro methods

In vitro methods have been used for nutritional evaluation of pig feeds for more than fifty years and during that time many different in vitro techniques have been developed estimating nutrient (including AA) digestibility.

In vitro methods can be divided into four groups [62]: i) dialysis cell methods; ii) pH-drop and pH-stat methods; iii) colorimetric methods; and iv) filtration methods; they can also be classified as simple methods in a closed system and complex methods. In vitro methods may comprise one-, two-, or three incubation steps and use different enzyme sources (industrial or natural, as digesta inocula and feces extracts).

The in vitro dialysis cells methods are based on the enzymatic digestion of protein with continuous removal of low-molecular-weight products by dialysis to prevent inhibition of enzymes activities by the end products of digestion. The first in vitro dialysis method included pre-incubation of the sample with pepsin for 30 min at 39 °C, followed by incubation with pancreatin with continuous stirring in the dialysis tube for 6 h [63]. The dialysis method and its modifications have been used to study the kinetics of enzymatic digestion of proteins and other nutrients [64–66]. The AID of protein and AA have been estimated by modified dialysis cells method described by Huang et al. [67]. Briefly, small amounts of samples were suspended in acid solution at pH 2.0 and digested with pepsin for 4 h at 37 °C. After adjusting pH to 7.6, the mixture were poured into dialysis tube containing phosphate buffer and trypsin and digested for 24 h at 37 °C with continuous stirring. Digestion products were collected from the external compartment by phosphate buffer circulation. Huang et al. [67] showed that the results obtained with this method could be affected by procedure condition, such as time of incubation, trypsin concentration, pH and the volume of incubation solution.

A significant correlations (*r* from 0.941 to 0.999) were found between in vitro digestibility of protein and AA determined by dialysis cell method and AID of protein and AA determined in pigs [67]. However, correlations were calculated only for four samples: rapeseed meal, cottonseed meal and two fish meals. These authors reported that the modified dialysis cells method could be recommended as sufficiently precise for estimation of ileal digestibility of protein and AA in pig feeds. However, this method is not routinely used in feed evaluation due to the high cost of dialysis tubes.

The pH-drop [68] and pH-stat [69] assays are other simple methods used to evaluate protein quality in processed feeds. These methods are based on monitoring the changes of pH after enzymatic digestion of feed protein. In the pH-drop method, pH is recorded after a 10-min sample incubation with enzymes (mixture of trypsin, chymotrypsin and peptidase) [68]. A close relationship (*r* = 0.90) was found between the pH after 10-min digestion in vitro and the ATTD of protein of 23 samples determined in rats [68]. The advantage of this method is a simple procedure, which takes no longer than 1 h. Authors mentioned that the pH-drop method can detect the effect of trypsin inhibitor, chlorogenic acid and heat treatment on protein digestibility. A high correlation between the results of pH-drop method and protein digestibility in rats was found only for plant proteins [68] and the equation derived did not accurately predict protein digestibility of meat and egg products. This method has been modified by adding additional 10-min incubation with proteinase from *Streptomyces griseus* [70].

The pH-stat method records the amount of alkali added to keep the pH constant for 10 min [69]. The consistency of pH-drop and pH-stat methods with the in vivo values measured in a great variety of feedstuffs and foods was poor [69, 71]. These authors suggested the application of different regression equations for each of feed in order to obtain a reliable estimate of protein digestibility. The pH-stat method was found to be highly reproducible in an interlaboratory study conducted by six different laboratories [72]. In the most of studies [69, 71] the results obtained with the pH-drop and pH-stat methods have been compared only with the results obtained in experiments with rats and only with total tract digestibility of protein, but not ileal digestibility. There is only one published study [73] comparing results of modified pH-stat method and SID of protein determined in pigs. Initial pH (*r* = 0.99) and degree of protein hydrolysis after 10 min (*r* = 0.96), but not after 120 min, were highly correlated with SID of protein for four samples of unprocessed and heat-treated soybean meal and rapeseed meal [73]. Both, pH-drop and pH-stat methods could provide a rapid information on protein damage of thermally processed feeds and foods. However, these methods have been used most often for monitoring of quality of thermally treated foods than feeds.

Colorimetric methods are primarily used mainly to predict starch digestibility or availability in processed feeds. The method consists of a two-step incubation with enzymes that liberate glucose or maltose. The concentration of sugars is determined after reaction with the reagents (e.g. anthrone, glucose oxidase-peroxidase, dinitrosalicylic acid) resulting in a light-absorbing products (5-hydroxymethylfurfural, quinonemine, aminonitrosalicyic acid, respectively), which is measured spectrophotometrically [74–76]. Some modifications of these methods seem to be very suitable to estimate starch availability in processed feeds.

Many different filtration methods have been developed and used mainly for the prediction of the total tract and ileal digestibility of nutrients. These methods consist of one, two or three sample incubations with enzymes in a closed system, followed by the collection of undigested residues using filtration (Table 7).

**Table 7:** In vitro filtration methods

Enzymes used in incubations			
1	2	3	References
One-step incubation methods:			
Pepsin			[77]
Trypsin			[78]
Papain			[79]
Pronase			[80]
Rennin			[81]
Duodenal digesta			[82]
Jejunal digesta			[82]
Feces extract			[82]
Two-step incubation methods:			
Pepsin	Pancreatin		[10, 83, 84]
Pepsin	Pronase		[85]
Pepsin	Trypsin		[86]
Pepsin	Jejunal fluid		[87]
Three-step incubations methods:			
Pepsin	Pancreatin	Cellulase	[93–95, 97, 98]
Pepsin	Pancreatin	Viscozyme	[96, 97]
Pepsin	Pancreatin	Rumen fluid	[99]

The first very simple in vitro method included only one-step incubation with pepsin [77]. This method and its modifications were used for monitoring the quality of heat-treated feeds. Other very simple in vitro methods using different proteases, such as trypsin [78], papain [79], pronase [80] or rennin [81] have also been proposed and successfully applied. A simple one-step incubation with three different inocula from duodenal digesta (12 h), jejunal digesta (48 h), or feces extract (48 h) was described in details by Löwgren et al. [82]. In vitro methods using inocula as a source of enzymes were used for measuring in vitro disappearance of various nutrients.

In the two-step incubation method, pepsin and pancreatin [10, 83, 84], pepsin and pronase [85], pepsin and trypsin [86] or pepsin and jejunal fluid [87] were applied to simulate ileal digestion of nutrients.

The AID values obtained using the two-step in vitro method based on incubations with pepsin and pancreatin according to Babinszky et al. [84] were closely related to the content of ileal digestible protein in seven feedstuffs and 16 diets determined in pigs (*r* = 0.99 and 0.95, respectively), but showed poor correlation with pig values for other 48 pig feeds (*R* ^2^ = 0.23) [88]. A close relationship was obtained only for five samples of wheat products (*R* ^2^ = 0.93), but not beans, peas, rapeseed products and soybean products (*R* ^2^ from 0.03 to 0.60) [88].

Among the two-step in vitro methods, the one developed by Boisen and Fernández [10] is most often used for the prediction of ileal digestibility of protein and AA in feedstuffs and diets for pigs. In this method, incubation with pepsin corresponding to digestion in the stomach is followed by incubation with pancreatin, simulating the digestion in the small intestine. Each incubation is performed at optimum pH, temperature and time. Undigested residues are collected by filtration, defatted with ethanol and acetone, and analyzed for dry matter and nitrogen contents. In vitro protein digestibility is calculated from the difference between the nitrogen content in the sample and undigested residues. The values of in vitro digestibility do not include any EPL, and thus they correspond to the TID of protein [9].

The TID of AA is calculated based on the TID of protein, since there is a close relationship between the TID of protein and TID of the majority of AA [10]. Equations describing the relationships between TID of protein and TID of individual AA for nine feedstuffs (barley, wheat, rye, oat, soybean meal, rapeseed meal, sunflower meal, grass meal and pea) are given in Table 8. The highest correlations were obtained for serine, histidine and tyrosine (*R* ^2^ = 0.95, 0.95 and 0.92, respectively) and the lowest for aspartic acid, proline and arginine (*R* ^2^ = 0.31, 0.56 and 0.57, respectively).

**Table 8:** Prediction equations of amino acids true ileal digestibility from protein true ileal digestibility [10]

Equations^a^	*R* ^2^
TID_IN VITRO_ Lys = 26.1 + 0.72 × TID_IN VITRO_ P	0.79
TID_IN VITRO_ Met = 9.9 + 0.91 × TID_IN VITRO_ P	0.83
TID_IN VITRO_ Cys = 24.0 + 0.72 × TID_IN VITRO_ P	0.60
TID_IN VITRO_ Thr = 25.5 + 0.71 × TID_IN VITRO_ P	0.82
TID_IN VITRO_ Ile = –1.4 + 1.01 × TID_IN VITRO_ P	0.85
TID_IN VITRO_ Arg = 46.1 + 0.52 × TID_IN VITRO_ P	0.57
TID_IN VITRO_ His = –5.1 + 1.06 × TID_IN VITRO_ P	0.95
TID_IN VITRO_ Leu = 19.8 + 0.78 × TID_IN VITRO_ P	0.84
TID_IN VITRO_ Phe = –1.1 + 1.01 × TID_IN VITRO_ P	0.88
TID_IN VITRO_ Val = 16.5 + 0.81 × TID_IN VITRO_ P	0.84

^a^TID_IN VITRO_ AA and TID_IN VITRO_ P – True ileal digestibility of individual amino acids and protein determined in vitro expressed in %; *Lys* Lysine, *Met* Methionine, *Cys* Cysteine, *Thr* Threonine, *Ile* Isoleucine, *Arg* Arginine, *His* Histidine, *Leu* Leucine, *Phe* Phenylalanine, *Val* Valine

*R* ^2^ - coefficient of determination

It has been shown that TID of protein determined in vitro was higher than AID of protein determined in pigs. The differences between TID and AID corresponded to total EPL. It has been reported that undigested dry matter (uDM) determined in vitro might be reliable indicator of total EPL calculated as difference between TID and AID of protein, since a close relationship between uDM and total EPL was obtained for 15 samples of single feedstuffs (*R* ^2^ = 0.61). This relation was described by following equation [10]:

$$ \text{total EPL} = 13.2+0066 \times \text{uDM} $$

where:

- total EPL – total endogenous protein losses expressed in g per kg dry matter intake,
- uDM – undigested dry matter determined in vitro expressed in g per kg dry matter.

Values obtained for meat and bone meal and barley hulls were deemed as outlayers and were not included in the regression.

The AID of protein and AA in vitro were predicted from TID_IN VITRO_ of protein by following equations according to Boisen and Férnandez [10]:

$$ \begin{array}{l}\mathrm{AI}{\mathrm{D}}_{\mathrm{IN}\ \mathrm{VITRO}}\mathrm{P}=\mathrm{TI}{\mathrm{D}}_{\mathrm{IN}\ \mathrm{VITRO}\mathrm{P}}-100\times \frac{13.2+0.066 \times \mathrm{uDM}}{\mathrm{P}\ \mathrm{content}}\\ {}\mathrm{AI}{\mathrm{D}}_{\mathrm{IN}\ \mathrm{VITRO}}\;\mathrm{AA}=\left(\mathrm{a}+\mathrm{b} \times \mathrm{TI}{\mathrm{D}}_{\mathrm{IN}\ \mathrm{VITRO}\mathrm{P}}\right)-100\\ {}\kern6em  \times \frac{\left(13.2+0.066\times \mathrm{uDM}\right)\times \mathrm{c}1\mathrm{AA}}{\mathrm{AA}\ \mathrm{content}}\end{array} $$

where:

- AID_IN VITRO_ P - apparent ileal digestibility of protein predicted in vitro expressed in %,
- AID_IN_ _VITRO_ AA – apparent ileal digestibility of individual amino acid predicted in vitro expressed in %,
- TID_IN VITRO_ P - true ileal digestibility of protein determined in vitro expressed in %,
- uDM – undigested dry matter determined in vitro expressed in g per kg of dry matter,
- P content – content of protein in feed expressed in g per kg of dry matter,
- (a + b × TID_IN VITRO_ P) – regression equation describing the relationship between true ileal digestibilty of protein determined in vitro and true ileal digestibility of individual amino acid determined in vitro; equations are given in Table 8,
- c1AA – the conversion factor from nitrogen to the individual amino acids in the total endogenous protein according to Boisen and Fernandez [10]: for lysine 0.0281, methionine 0.0079, cysteine 0.0157, threonine 0.0413, isoleucine 0.0242, leucine 0.0393, histidine 0.0106, phenylalanine 0.0285, tyrosine 0.0217, valine 0.0345, arginine 0.0224, alanine 0.0402, aspartic acid 0.0795, glutamic acid 0.0999, glycine 0.0655, proline 0.0620, serine 0.0411,
- AA content – content of individual amino acid in feed expressed in g per kg of dry matter.

The two-step in vitro method developed by Boisen and Fernández [10] was validated with 48 diets with known AID of protein and AA determined in pigs. The relationship for protein was considerably low (*R* ^2^ = 0.57), which was partly due to the narrow variation range in AID of protein determined in pigs [10]. The correlation between AID of AA measured in pigs and estimated in vitro was generally higher for essential AA and lower for non-essential AA than for protein [10]. A close relationship between the AID of AA determined in pigs and method of Boisen and Fernández [10] was found by Święch and Buraczewska [89] and Cho and Kim [90]. Święch and Buraczewska [89] compared AID determined in pigs and predicted using in vitro method in 12 diets containing faba bean, pea or lupin mixed with casein as a protein source. The relationship between AID of protein determined in pigs and predicted in vitro was close (*R* ^2^ = 0.90). The correlation between in vivo and predicted in vitro values of AA AID was the highest for cysteine and methionine (*R* ^2^ = 0.94 and 0.89, respectively), whereas lower for lysine (*R* ^2^ = 0.76) and poor for threonine (*R* ^2^ = 0.43) [89]. Cho and Kim [90] compared AID of protein and AA determined in pigs and predicted in vitro in ten nursery pig diets. The highest correlation was found for glycine, isoleucine and threonine (*R* ^2^ = 0.89, 0.85 and 0.83, respectively) and the lowest for proline, tyrosine and alanine (*R* ^2^ = 0.24, 0.35 and 0.40, respectively).

The SID of protein and AA in pigs were predicted from TID_IN VITRO_ of protein by following equations according to Boisen [9]:

$$ \begin{array}{l}\mathrm{SID}\;\mathrm{P}=\frac{\frac{\mathrm{P}\ \mathrm{content}\times \mathrm{TI}{\mathrm{D}}_{\mathrm{IN}\ \mathrm{VITROP}}}{100}-0.0106\times \mathrm{uDM}}{\mathrm{P}\ \mathrm{content}}\times 100\\ {}\mathrm{SID}\ \mathrm{AA}=\frac{\frac{\mathrm{AA}\ \mathrm{content}\times \mathrm{TI}{\mathrm{D}}_{\mathrm{IN}\ \mathrm{VITROP}}\ }{100}-\mathrm{c}2\mathrm{AA} \times 0.0106\times \mathrm{uDM}}{\mathrm{AA}\ \mathrm{content}}\times 100\ \end{array} $$

where:

- TID_IN VITRO_ P - true ileal digestibility of protein determined in vitro expressed in %,
- uDM – undigested dry matter determined in vitro expressed in g per kg dry matter,
- c2AA – conversion factor from nitrogen to AA in extra endogenous protein according to Boisen and Moughan [6]: for lysine 0.188, methionine 0.063, threonine 0.281, tryptophan 0.075, isoleucine 0.156, leucine 0.250, histidine 0.094, phenylalanine 0.188, tyrosine 0.125, valine 0.219,
- P content – protein content in feed expressed in g per kg of dry matter,
- AA content – amino acid content in feed expressed in g per kg of dry matter.

A close relationship between the SID of AA determined in pigs and the two-step in vitro method of Boisen and Fernandez [10] was confirmed by Święch [91] and Jezierny et al. [92]. The SID values of protein and AA of seven feedstuffs (after exclusion of raw soybean) determined in pigs and in vitro were closely related [91], the highest relationships were found for protein, phenylalanine and valine (*R* ^2^ = 0.937, 0.925 and 0.918, respectively) and the lowest for threonine (*R* ^2^ = 0.796). Only relationship for methionine was poor and not significant (*R* ^2^ = 0.477). Similar results have been obtained by Jezierny et al. [92], who found high correlations between SID of protein and AA of grain legumes determined in pigs and predicted in vitro. The highest relationship was obtained for tryptophan, cysteine and histidine (*R* ^2^ = 0.91, 0.91 and 0.89, respectively) and the lowest for lysine (*R* ^2^ = 0.73). It seems that the in vitro method may not be suitable for all types of feedstuffs, because it does not reflect effects of trypsin inhibitor content [28]. In contrast, the in vitro SID of AA is close related to tannin content [28, 92].

It can be concluded that the two-step in vitro method developed by Boisen and Fernández [10] may be use for estimation of SID of protein and AA in pig feeds with exception of feeds containing trypsin inhibitor. However, further studies comprising evaluation of differently treated feeds would be needed.

In vitro three-step methods were developed mainly for predicting nutrients digestibility in the whole digestive tract. They involve consecutive incubations of feed samples with enzymes simulating digestion in stomach, small intestine and large intestine, such as pepsin, pancreatin and fiber-degrading enzymes [93–98] or pepsin, pancreatin and rumen fluid [99]. Cellulase [93–95, 97, 98] and multi-enzyme viscozyme complex containing arabinose, cellulase, β-glucanase, hemicellulase, xylanase, and pectinase [96, 97] were used as fiber-degrading enzymes.

Among the three-step incubation in vitro methods, the one developed by Boisen and Fernández [96] was the most thoroughly tested and verified as the basis for ATTD of energy in pigs and feed energy values. In this method, sample incubation with pepsin is followed by incubations with pancreatin and with multi-enzyme complex viscozyme. Each incubation is performed at the optimum pH, temperature and time. Undigested residues are collected by filtration, defatted with ethanol and acetone, and analyzed for dry matter and ash contents. In vitro digestibility of organic matter is calculated from the difference between the content of organic matter in the sample and undigested residues.

A set of equations relating the in vitro digestibility of organic matter of a wide range of feeds to the ATTD of energy determined in pigs has been developed by Boisen and Fernández [96] (Table 9). When all 33 evaluated feedstuffs were included, the relationship was not satisfactory (Equation No 1; *R* ^2^ = 0.69). Elimination of raw potato starch and meat and bone meal improved greatly this relationship (Equation No 2; *R* ^2^ = 0.94), whereas further elimination of potato protein concentrate, sugar beet pulp and dried whey resulted in a rather small improvement (Equation No 3; *R* ^2^ = 0.96). Therefore, the Equation no 2 has been recommended by the authors for practical energy feed evaluation. This equation was validated using 34 feed mixtures (*R* ^2^ = 0.87) and the possibility of application of the in vitro digestible organic matter as the basis of estimation of ATTD of energy and DE in pig diets was confirmed [96]. The ME and NE can be calculated from the DE content using equations (Table 10) provided by Boisen [100]

**Table 9:** Prediction equations of energy apparent total tract digestibility from in vitro organic matter digestibility [96]

No^a^	Equation^b^	n	*R* ^2^
1	ATTD_IN VITRO_ E = 4.8 + 0.881 × D_IN VITRO_ OM	33	0.69
2	ATTD_IN VITRO_ E = –14.0 + 1.106 × D_IN VITRO_ OM	31	0.94
3	ATTD_IN VITRO_ E = –14.7 + 1.117 × D_IN VITRO_ OM	28	0.96

^a^Equation: No. 1 - estimated for all feedstuffs; No. 2 – estimated after exclusion of values for raw potato starch and meat and bone meal; No. 3 - estimated after exclusion of values for raw potato starch, meat and bone meal, potato protein concentrate, sugar beet pulp and dried whey;

^b^ATTD_IN VITRO_ E – Apparent total tract digestibility of energy predicted in vitro expressed in %; D_IN VITRO_ OM – Digestibility of organic matter determined in vitro expressed in %

*R* ^2^ - coefficient of determination;

**Table 10:** Title: calculation of energy values of pig feeds using in vitro organic matter digestibility [100]

Calculation of energy values of pig feeds	
1. Gross energy (GE, MJ/kg of dry matter)	
2. Digestible energy (DE, MJ/kg of dry matter)	
DE = (GE × 1.106 × D_IN VITRO_ OM – 14.0)/100	
D_IN VITRO_ OM: In vitro digestibility of organic matter expressed in %	
3. Metabolizable energy (ME, MJ/kg of dry matter)	
ME = DE – 0.17 × N	
N: Nitrogen content in feed expressed in % of dry matter	
4. Net energy (NE, MJ/kg of dry matter)	
NE = DE × 0.75 – 1.88	

Concordance of feed energy values determined in pigs and using the three-step in vitro method described by Boisen and Fernández [96] was confirmed by Święch and Buraczewska [101] and Noblet and Juguelin-Peyraud [102].

The in vitro organic matter digestibility is affected by incubation conditions such as particle size, sample weight and stirring [96]. The increase of particle size (from 1 to 3 mm) and increase of sample weight (from 0.5 to 1.0 g of some high-protein feeds) reduced the in vitro digestibility of organic matter, whereas continuous stirring appeared to be necessary during incubation of some starch-rich feedstuffs as maize, tapioca and peas and less important for digestion of wheat and barley.

Modification of the Boisen and Fernández method [96] consisting in replacing viscozyme by cellulase gave satisfactory results of evaluation of different barleys and barley mixtures [93–95], but decreased accuracy of predicting the ATTD of energy of wheat [97].

Other interesting in vitro technique is a gastrointestinal dynamic in vitro model, which mimics the processes going on in the stomach and small intestine (TIM-1) [103] and in the large intestine (TIM-2) [104] of pigs and humans. The computer-controlled TIM-1 model simulates gastric pH change, peristaltic movements, gastric emptying rates, intestinal transit times, enzyme secretion and small intestinal absorption [103]. It comprises digestion chambers, peristaltic pump, pH electrodes, filters, and water bath. The TIM-2 model is complementary to the TIM-1 and simulates removal of fermentation products and water with peristaltic mixing to obtain and handle physiological concentration of microorganism, dry matter and microbial metabolites [104]. Both systems are programmed to simulate physiological conditions in the gastrointestinal tract of pigs based on physiological values obtained in vivo. Meunier et al. [105] reported that the dynamic model may be used to estimate protein ileal digestibility;, however, it cannot be used to predict the protein and energy values of feeds differing in fiber contents. It seems that the dynamic in vitro model is a more complex, high cost system and may be an alternative for physiological studies of gastrointestinal tract in pigs.

Nutritional values of pig feeds may be also predicted using near-infrared reflectance spectroscopy (NIRS), which is a rapid, non-destructive and relatively inexpensive technique. It has been used routinely in feed industry for determination of chemical composition including AA content [106–109]. Possibility of predicting energy value of feedstuffs have been also investigated [49, 110–113] by NIRS. However, results of some studies have been not satisfactory due to the low number of samples used for calibration and relatively low variability between samples [49, 111, 112]. There have been only few published studies comparing NIRS values and ileal digestibility of protein and AA determined on pigs [112, 114]. However, in these studies calibration of NIRS was done using the predicted ileal digestibility of protein and AA determined by in vitro method described by Boisen and Fernández [10]. The NIRS method is promising, but till now it is not sufficiently validated.

### General remarks and perspectives

The use and choice of alternative methods may depend on the actual demands of feed industry and systems of pig nutrition. Also, the availability and cost of necessary materials as laboratory equipment and animal test regulations are important factors. Up to now, the alternative methods of energy evaluation based on their chemical composition and in common use in swine production. Rat tests may become necessary for determination of the effects of feed processing on both energy and protein availability. Since protein is the most costly and deficient nutrient, its proper evaluation is primordial for effective swine production. Therefore, the alternative methods of protein evaluation comprising ileal digestible amino acid content should be more widely applied.

## Conclusions

The use of alternative methods of feed evaluation is an important way of reduction of stressful animal experiments. Dietary concentration of energy available to pigs as digestible and metabolizable energy is estimated with satisfactory precision from chemical composition of feed using equations comprising different feed components. This method is commonly used in practice. Energy value of feeds may be determined in rats as animal model with good precision for feeds with moderate fiber and fat content. It may be also assayed as digestible energy by three-step in vitro method simulating digestion of nutrients in the whole digestive tract and recalculated using validated equations according to Boisen and Fernández [96] and Boisen [100]. Feed protein evaluation is presently based on determination of ileal digestibility of essential amino acids in cannulated pigs. The procedure can be replaced by measurement performed in vivo in rats or assayed by the two-step in vitro method simulating digestion of protein up to the end of the small intestine. The experimental values can be recalculated to standardized ileal digestible amino acids according to equations developed by Boisen [9].

## Abbreviations

AA: Amino acid
AID: Apparent ileal digestibility
ATTD: Apparent total tract digestibility
DE: Digestible energy
EAAL: Endogenous amino acids losses
EPL: Endogenous protein losses
ME: Metbolizable energy
MNBT: Mobile nylon bag technique
NE: Net energy
NIRS: Near-infrared reflectance spectroscopy
SID: Standardized ileal digestibility
TID: True ileal digestibility
TIM-1: In vitro model of the stomach and small intestine
TIM-2: In vitro model of the large intestine
uDM: Undigested dry matter determined in vitro
